# Prognostic model integrating histology, systemic inflammation, and recurrence status predicts immunotherapy response in advanced non-small-cell lung cancer patients

**DOI:** 10.1186/s13062-025-00674-3

**Published:** 2025-07-03

**Authors:** F. V. Moiseenko, M. A. Krasavina, I. R. Agranov, E. V. Artemieva, A. P. Oganesian, A. S. Gabina, M. L. Makarkina, E. O. Elsakova, V. A. Henshtein, N. M. Volkov, V. V. Egorenkov, V. M. Moiseenko, M. Yu. Fedyanin, G. S. Kopeina, B. Zhivotovsky, A. V. Zamaraev

**Affiliations:** 1 N.P. Napalkov St. Petersburg City Cancer Hospital, St. Petersburg, Russia; 2 N.N. Petrov Institute of Oncology, St. Petersburg, Russia; 3 I.I Mechnikov North-Western State Medical University, St. Petersburg, Russia; 4 Moscow Medical City Hospital “Kommunarka”, Moscow, Russia; 5 NN Blokhin National Medical Research Center of Oncology, Moscow, Russia; 6 N.I. Pirogov National Medical and Surgical Center, Moscow, Russia; 7 Engelhardt Institute of Molecular Biology, Russian Academy of Sciences, Moscow, Russia; 8 Faculty of Medicine, MV Lomonosov Moscow State University, Moscow, Russia; 9 Institute of Environmental Medicine, Karolinska Institute, Box 210, Stockholm, Sweden

**Keywords:** Prognostic model, Non-small-cell lung cancer, Immunotherapy, NLR

## Abstract

**Background:**

Non-small-cell lung cancer (NSCLC) exhibits variable outcomes and remains a leading cause of cancer-related mortality, despite advances in immunotherapy. This study aimed to develop a prognostic model using real-world data (RWD) to stratify patients by survival outcomes and evaluate the benefit of immunotherapy across risk groups.

**Methods:**

A retrospective cohort of 270 patients with NSCLC (2015–2024) treated with chemotherapy alone (54%) or chemoimmunotherapy (46%) was analyzed. Clinical, laboratory (neutrophil-to-lymphocyte ratio [NLR], platelet-to-lymphocyte ratio [PLR], monocyte-to-lymphocyte ratio [MLR]), and histopathological data were collected. Multivariate Cox regression identified prognostic factors for overall survival (OS) and validated them via bootstrapping.

**Results:**

The cohort (median age, 65; 78% male) had a median OS of 11.2 months and a median progression-free survival (PFS) of 7.7 months. The final prognostic model incorporated histology (adenocarcinoma vs. large cell/squamous cell carcinoma/rare subtypes: HR = 1.6–2.03), recurrence state (HR = 0.51), and NLR (HR = 1.13). Patients were stratified into low- (median OS = 14.6 months) and high-risk (median OS = 9.6 months; *p <* 0.001) groups. Immunotherapy significantly increased PFS in low-risk patients (12.2 vs. 7.1 months, *p =* 0.002) and showed an increasing trend in OS (16.9 vs. 11.3 months, *p =* 0.12). High-risk patients derived no OS/PFS benefit (*p* ≥ 0.56).

**Conclusion:**

This RWD-derived prognostic model effectively stratifies NSCLC patients into distinct risk groups. Immunotherapy-chemotherapy provided meaningful PFS improvement in low-risk patients but minimal benefit in high-risk subgroups, underscoring the need for tailored therapeutic strategies.

**Supplementary Information:**

The online version contains supplementary material available at 10.1186/s13062-025-00674-3.

## Introduction

Non-small-cell lung cancer (NSCLC) represents the most prevalent form of solid tumor malignancy in adults, as well as the leading cause of cancer-related mortality worldwide. Despite paradigm-shifting advances in therapeutic strategies, including immunotherapy, targeted therapies, and anti-angiogenic agents, clinical outcomes remain heterogeneous. Numerous clinical factors are correlated with the behavior, clinical course duration, and response to conventional treatment of this disease. These include performance status, appetite and weight loss, serum biomarkers or metabolic markers, and mutational and phenotypic profiles [1, 2]. Multiple prognostic scales have been successfully developed for solid tumors, each one incorporating a different subset of these factors. Some such scales are routinely used to drive treatment decisions. For instance, multivariable risk stratification systems, such as the Memorial Sloan Kettering Cancer Center (MSKCC) criteria [3] for renal cell carcinoma and the Genito-Urinary Radiation Oncologists of Canada (GUROC) system [4] for prostate cancer, have revolutionized personalized oncology care, but no analogous framework has achieved widespread adoption in thoracic oncology.

The advent of immunotherapy has fundamentally transformed and continues to evolve the treatment paradigm for NSCLC. Landmark clinical trials have demonstrated significant survival benefits across NSCLC subgroups, including pembrolizumab in KEYNOTE-189 and KEYNOTE-024, atezolizumab in IMpower150, and nivolumab-ipilimumab in CheckMate 227 [5–8]. All these studies support the administration of immunotherapy for most NSCLC patients in the first-line setting. However, the magnitude of the clinical benefit might vary greatly across groups. Beyond PD-L1 expression, many emerging biomarkers, such as smoking history, tumor mutational burden (TMB), *POLE/POLD* mutations, and systemic inflammation markers, are under investigation [9–11]. Despite these efforts, no single biomarker reliably predicts immunotherapy response with sufficient accuracy [12].

This study addresses the above-mentioned critical gaps by developing and validating a novel prognostic model derived from real-world data (RWD) encompassing clinical, laboratory, and histopathological variables. We evaluated the differential survival benefits of immunotherapy-chemotherapy combinations across prognostic subgroups by stratifying patients into distinct risk categories. Our approach builds upon emerging evidence that integrative models combining tumor histology (adenocarcinoma vs. large cell carcinoma), systemic inflammation indices, and recurrence status may outperform single-biomarker strategies in predicting immunotherapy responsiveness. The resulting framework aims to optimize therapeutic selection, mitigate unnecessary treatment-related morbidity, and allocate healthcare resources more efficiently.

## Materials and methods

### Data collection

**Fig. 1:**
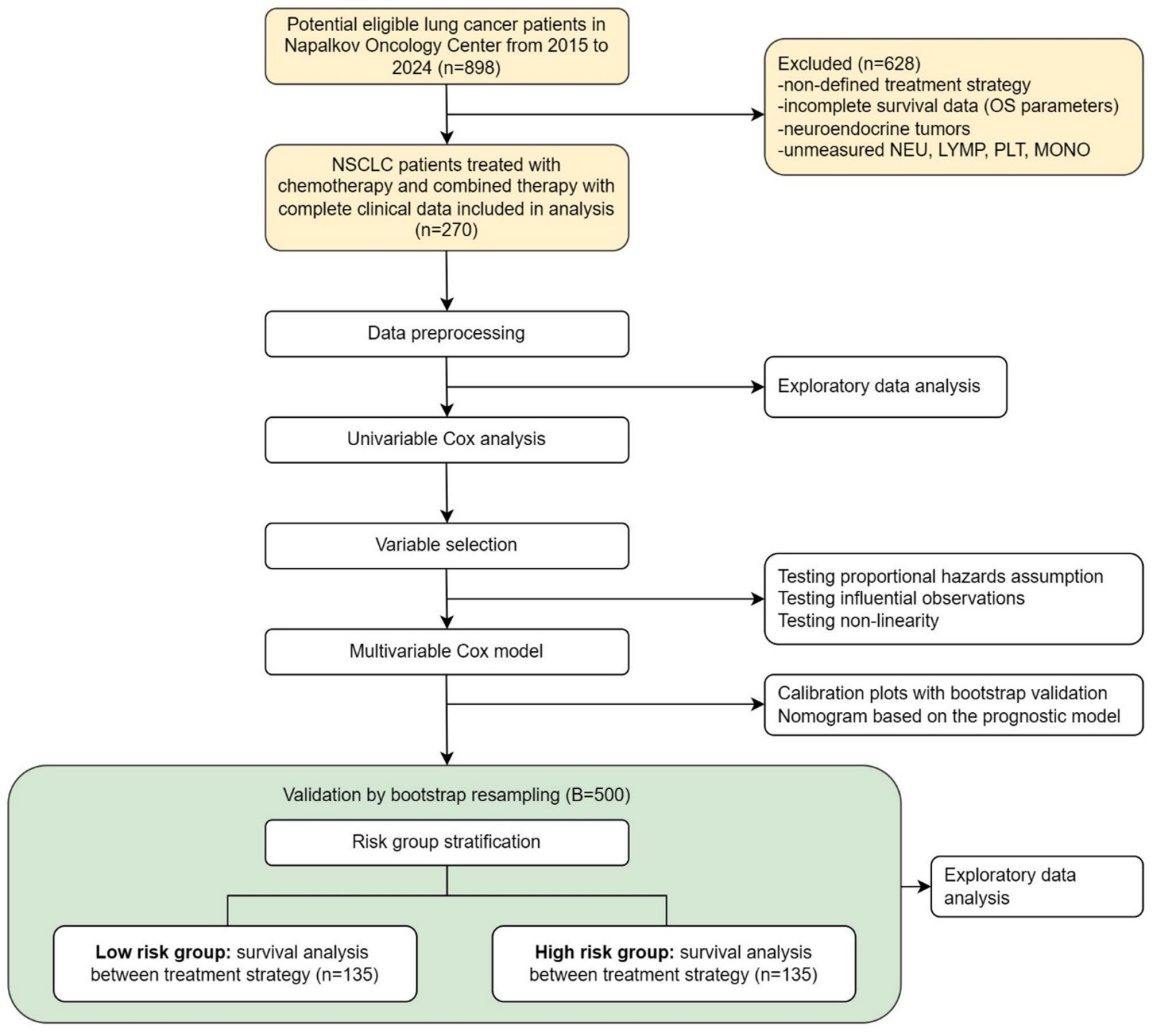
Flowchart of data analysis and patient risk group stratification. The flowchart outlines a survival analysis process for non-small-cell lung cancer (NSCLC) patients at CRPCSTMC (2015–2024). It begins with cohort identification, including patients treated with chemotherapy/combined therapy, excluding those with undefined strategies, incomplete survival data, neuroendocrine tumors, or missing biomarkers: neutrophils (NEU), lymphocytes (LYMP), platelets (PLT), and monocytes (MONO). Data preprocessing and exploratory analysis are followed by univariable Cox regression to screen prognostic variables. Variable selection involves testing proportional hazards assumptions, influential observations, and non-linearity. A multivariable Cox model is then built, validated via bootstrap calibration plots, and visualized as a nomogram. Patients are stratified into low- and high-risk groups (*n* = 135 each), with survival analysis comparing treatment strategies between groups. The stratification and survival analysis are validated by bootstrap resampling of the study dataset (B = 500)

This retrospective study included 270 patients with non-small-cell lung cancer (NSCLC) from a database of N.P. Napalkov St. Petersburg Clinical Research and Practical Center of Specialized Types of Medical Care (CRPCSTMC, Oncological) between 2015 and 2024. The inclusion criteria were a histologically confirmed NSCLC diagnosis and availability of complete clinical data (demographics, TNM stage, treatment history, laboratory parameters) (Fig. 1). Patients received either chemotherapy alone (*n* = 147, 54%) or a combination of immunotherapy and chemotherapy (*n* = 123, 46%). All treatment decisions were reviewed by the thoracic multidisciplinary team and documented. TNM staging was performed according to the AJCC 8th edition. Treatment responses were evaluated using RECIST 1.1. The study protocol was approved by the Ethics Committee of the CRPCSTMC (Approval No. 4 from 14.03.2023). Informed consent was waived due to the retrospective nature of the study design. Demographic and clinical characteristics collected included gender, age, body mass index (BMI), tumor histology, TNM stage, recurrence status, previous treatment, radiotherapy, and surgical intervention. Laboratory parameters included the neutrophil-to-lymphocyte ratio (NLR), monocyte-to-lymphocyte ratio (MLR), and platelet-to-lymphocyte ratio (PLR). These ratios were calculated from complete blood count results obtained prior to treatment initiation. Survival data were anonymized and extracted from medical records as well as follow-up calls. The primary endpoints were overall survival (OS) and progression-free survival (PFS). OS was defined as the time from treatment initiation to death from any cause or last follow-up. PFS was defined as the time from treatment initiation to disease progression or death. The study was conducted according to the flowchart presented in Fig. 1.

### Statistical analysis

Descriptive statistics were presented as medians and interquartile ranges for continuous variables, and absolute numbers and percentages for categorical variables. Survival was estimated using the Kaplan-Meier method. Log-rank tests were used to compare the analyzed groups.

### Univariate and multivariate Cox regression analysis

Univariate Cox proportional hazards models were first constructed to identify potential prognostic factors for OS and predictive factors for PFS. HR and 95% confidence intervals (CI) were calculated. Variables with *p* < 0.1 in univariate analysis were included in the multivariate model. The final multivariate model for OS was obtained using backward stepwise selection with the Akaike Information Criterion (AIC) as the stopping rule. The resulting multivariate model was as follows: *OS ~ Histology + Recurrence status + NLR*.

### Assessing the validity of the multivariate Cox model

To evaluate potential non-linear associations between continuous predictors and OS, restricted cubic splines (RCS) with 3–4 knots were tested for continuous predictors. Likelihood ratio tests (LRT) assessed whether spline models significantly improved fit over linear models. Results were visualized as predicted log-hazard curves with 95% confidence intervals. The proportional hazards assumption for the final multivariate Cox model was assessed using Schoenfeld residuals. Plots of scaled Schoenfeld residuals were examined for trends, and a global test was performed to assess the overall validity of the proportional hazards’ assumption. Deviance residuals were also plotted to identify outliers or influential observations.

### Model calibration and nomogram development

The multivariate Cox model was internally validated using bootstrap resampling (500 iterations). The model’s discriminative ability was assessed using Harrell’s C-index. Calibration was evaluated by comparing predicted and observed survival probabilities at 12 and 24 months using calibration plots. Calibration curves were generated to assess the agreement between predicted and observed survival probabilities at 12 and 24 months. Model calibration was assessed using bootstrapping, providing bias-corrected estimates. A nomogram was constructed based on the final multivariate Cox model to provide a graphical representation of the prognostic factors and their impact on survival probability. The nomogram was created using the ‘rms’ package in R.

### Risk stratification

Patients were stratified into low- and high-risk groups based on the median linear predictor score from the multivariate Cox model. The linear predictor was calculated as follows: *LP = β*_*1*_*(Histology) + β*_*2*_*​(Recurrence status) + β*_*3*_*​(NLR)*, where *β₁*,* β₂*,* β₃*, are the estimated coefficients from the multivariate Cox model. Patients were classified as low-risk or high-risk based on whether their linear predictor value was below or above the median, respectively. Kaplan-Meier curves were generated for these risk groups, and the log-rank test was used to compare survival between groups.

### Bootstrap validation and aggregated survival curves

To assess the stability of the risk stratification and the effect of treatment (chemotherapy alone vs. immunotherapy plus chemotherapy), bootstrap aggregated survival curves were generated. This involved creating 500 bootstrap samples, stratifying patients into risk groups, and aggregating the resulting survival curves for risk groups or treatments in each risk group. To evaluate the stability of our findings, the proportion of significant log-rank tests (*p* < 0.05) across 500 bootstrap samples was calculated. This approach provides insight into the consistency of observed risk group differences across resampled datasets and the treatment effect difference in each risk group. The 95% confidence interval for this proportion was calculated using the Wilson method, offering a range of plausible values for the true proportion of significant results. The interval-specific comparisons between treatment chemotherapy alone and immunotherapy plus chemotherapy for the risk groups using bootstrap approaches were also performed. This time-dependent analysis (6, 9, 12, 18, 21, and 24 months) allows the assessment of treatment differences at various time points throughout the follow-up period. The 500 bootstrap samples were used to estimate the significance of treatment differences at each time point, providing a more precise understanding of when treatment effects may emerge or change over time.

### Software

All statistical analyses were conducted using R version 4.1.0. The “*survival*,” “*survminer*,” “*rms*,” “*ggplot2*,” “*purrr*,” and “*binom*” packages were used for survival analysis, visualization, nomogram creation, data manipulation, and confidence interval calculation. Clinical data tables and figures were generated using OncoPredict v1.0 (Certificate of Software Registration № RU2023615486). The significance level was set at *p* < 0.05 for all analyses.

## Results

### The baseline clinicopathological characteristics of NSCLC patients enrolled in the study

The study cohort comprised 270 patients with NSCLC, predominantly male (78%), with a median age of 65 years (IQR: 59–71). Most patients presented with advanced disease: 63% had stage IV cancer, and 46% had T4 tumors. Histologically, adenocarcinoma (33%), squamous cell carcinoma (26%), and large cell carcinoma (29%) were the most common subtypes. Patients received either chemotherapy alone (54%) or a combination of chemotherapy and checkpoint inhibitors (46%). Systemic inflammation markers included a median neutrophil-to-lymphocyte ratio (NLR) of 2.99 (IQR: 2.13–3.99), platelet-to-lymphocyte ratio (PLR) of 166 (IQR: 118–223), and monocyte-to-lymphocyte ratio (MLR) of 0.42 (IQR: 0.31–0.59) (Table 1).

**Table 1:** The clinicopathological characteristics of 270 patients with non-small-cell lung cancer. Histology: LUAD (lung adenocarcinoma), LUSC (squamous cell carcinoma), LCLC (large cell lung cancer). TNM (T/N/M): tumor, node, and metastasis staging classifications. “X” denotes unspecified staging. RT: radiotherapy. NLR/MLR/PLR: Neutrophil-to-lymphocyte, monocyte-to-lymphocyte, and platelet-to-lymphocyte ratios (continuous variables)

Characteristic	*N* = 270^*1*^
**Gender**	
female	59 (22%)
male	211 (78%)
**Age**	65 (59, 71)
**BMI**	24.7 (22.0, 27.9)
**Histology**	
LUAD	89 (33%)
LUSC	71 (26%)
LCLC	78 (29%)
other	32 (12%)
**TNM (T)**	
1	23 (8.5%)
2	48 (18%)
3	66 (24%)
4	124 (46%)
X	9 (3.3%)
**TNM (N)**	
0	38 (14%)
1	54 (20%)
2	105 (39%)
3	58 (21%)
X	15 (5.6%)
**TNM (M)**	
0	100 (37%)
1	164 (61%)
X	6 (2.2%)
**Stage_groups**	
I-II	19 (7.0%)
III	82 (30%)
IV	169 (63%)
**Recurrence status**	
De novo	227 (84%)
Recurrent	43 (16%)
**NLR**	2.99 (2.13, 3.99)
**MLR**	0.42 (0.31, 0.59)
**PLR**	166 (118, 223)
**Treatment**	
Chemo	147 (54%)
ICI + Chemo	123 (46%)
**RT**	
no	240 (89%)
yes	30 (11%)
**Surgery**	
no	264 (98%)
yes	6 (2.2%)

^*1*^n (%); Median (Q1, Q3)

**Fig. 2:**
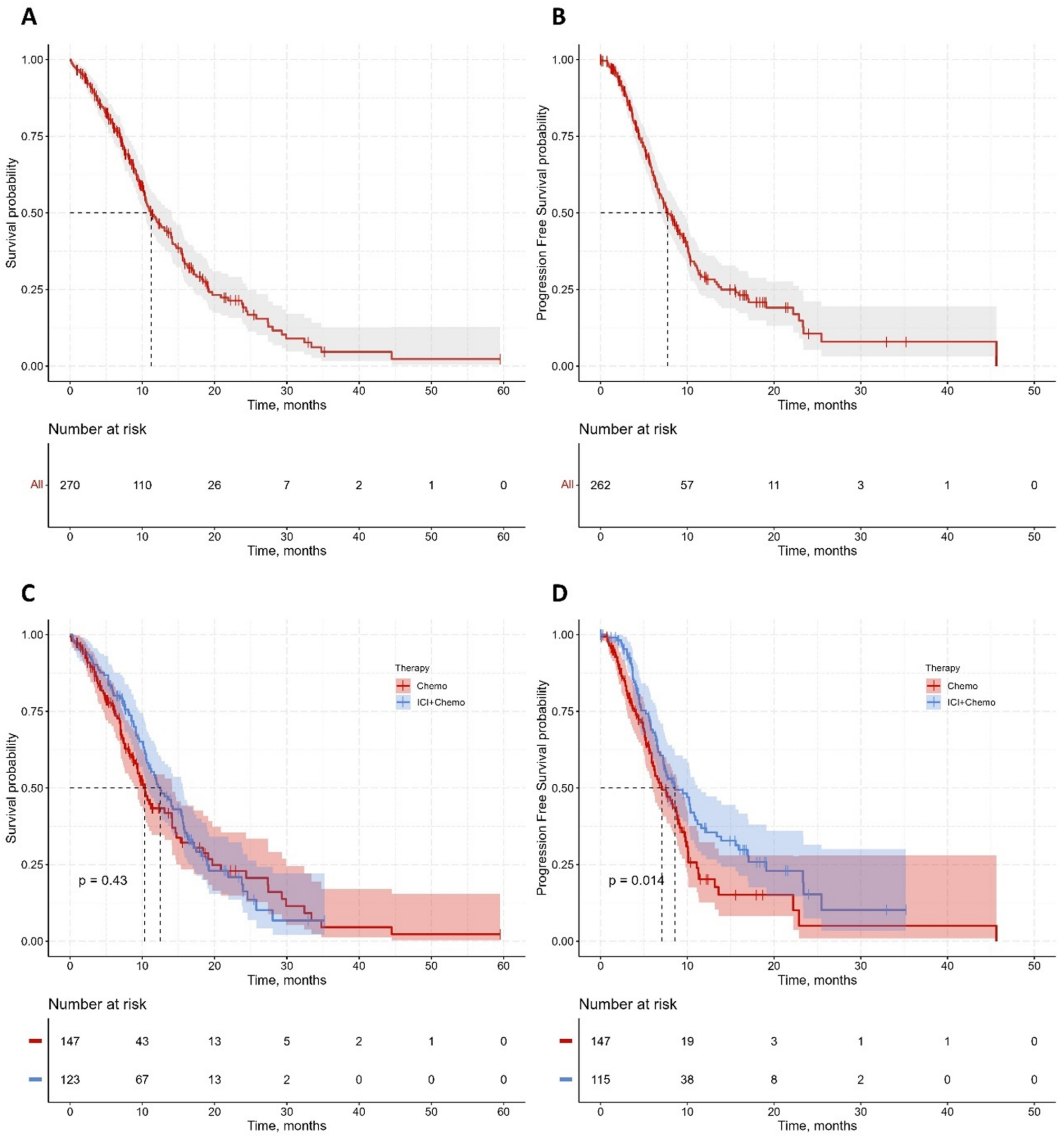
Kaplan-Meier survival analysis of NSCLC patients. **(A)** Overall survival (OS) for the entire cohort. **(B)** Progression-free survival (PFS) for the entire cohort. **(C)** OS stratified by treatment group: Chemotherapy (red) vs. Chemotherapy + Immune Checkpoint Inhibitors (ICI) (blue). **(D)** PFS stratified by treatment group: Chemotherapy (red) vs. Chemotherapy + ICI (blue). Numbers at risk at specified time points are listed below each panel. Red and blue “+” symbols denote censored data. Log-rank test *P* values are reported for treatment comparisons (C and D)

The median OS for the entire cohort was 11.2 months (95% CI: 10.3–14.0), with a median progression-free survival (PFS) of 7.73 months (95% CI: 6.7–9.47) (Fig. 2A, B). Immunotherapy plus chemotherapy demonstrated a non-significant trend towards improved patient OS (12.5 vs. 10.3 months, *p =* 0.43), while it significantly prolonged PFS (8.6 vs. 7.1 months, *p =* 0.014). (Fig. 2C, D).

### Univariate and multivariate Cox regression models

Univariate Cox regression analysis identified several significant prognostic factors for OS. Patients with large cell carcinoma (HR = 1.82, *p* = 0.003) or rare histological subtypes (HR = 1.89, *p* = 0.012) exhibited nearly double the mortality risk compared to patients with adenocarcinoma, while recurrence (HR = 0.49, *p* = 0.012) and lower NLR (HR = 1.12, *p* = 0.01) showed a positive correlation with OS (Table 2).

**Table 2:** Univariate Cox regression analysis of overall survival (OS) and progression-free survival (PFS) in NSCLC patients. Histology: LUAD (lung adenocarcinoma), LUSC (squamous cell carcinoma), LCLC (large cell lung cancer). TNM (T/N/M): tumor, node, and metastasis staging classifications. “X” denotes unspecified staging. RT: radiotherapy. NLR/MLR/PLR: Neutrophil-to-lymphocyte, monocyte-to-lymphocyte, and platelet-to-lymphocyte ratios (continuous variables)

	Univariate - OS				Univariate - PFS			
Characteristic	*N*	HR	95% CI	*p*-value	*N*	HR	95% CI	*p*-value
**Gender**								
female	59	—	—		57	—	—	
male	211	1.18	0.80, 1.74	0.4	207	1.18	0.81, 1.73	0.4
**Age**	270	1.00	0.99, 1.02	0.8	264	1.00	0.98, 1.02	0.8
**BMI**	270	0.98	0.95, 1.01	0.2	264	0.98	0.95, 1.02	0.3
**Histology**								
LUAD	89	—	—		86	—	—	
LUSC	71	1.36	0.90, 2.06	0.14	69	1.23	0.81, 1.87	0.3
LCLC	78	1.82	1.22, 2.69	0.003	77	1.41	0.95, 2.10	0.087
other	32	1.89	1.15, 3.10	0.012	32	1.01	0.56, 1.83	> 0.9
**TNM (T)**								
1	23	—	—		23	—	—	
2	48	0.73	0.38, 1.40	0.3	48	0.70	0.37, 1.33	0.3
3	66	1.06	0.57, 1.97	0.8	65	1.08	0.60, 1.96	0.8
4	124	1.23	0.70, 2.17	0.5	120	0.92	0.53, 1.62	0.8
X	9	0.88	0.32, 2.46	0.8	8	1.29	0.50, 3.34	0.6
**TNM (N)**								
0	38	—	—		37	—	—	
1	54	1.55	0.88, 2.71	0.13	53	1.32	0.72, 2.42	0.4
2	105	1.59	0.97, 2.63	0.068	102	1.57	0.91, 2.70	0.10
3	58	1.63	0.95, 2.81	0.076	57	1.90	1.07, 3.37	0.029
X	15	1.22	0.57, 2.61	0.6	15	1.80	0.84, 3.84	0.13
**TNM (M)**								
0	100	—	—		98	—	—	
1	164	1.11	0.80, 1.55	0.5	160	1.15	0.82, 1.62	0.4
X	6	0.66	0.20, 2.22	0.5	6	0.47	0.11, 1.94	0.3
**Stage groups**								
I-II	19	—	—		19	—	—	
III	82	2.62	1.17, 5.90	0.020	80	1.50	0.73, 3.09	0.3
IV	169	2.58	1.20, 5.56	0.016	165	1.66	0.83, 3.29	0.2
**Recurrence status**								
De novo	227	—	—		221	—	—	
Recurrent	43	0.49	0.29, 0.86	0.012	43	0.74	0.46, 1.20	0.2
**NLR**	270	1.12	1.03, 1.21	0.010	264	1.08	0.98, 1.19	0.12
**MLR**	270	1.23	0.70, 2.15	0.5	264	1.29	0.63, 2.66	0.5
**PLR**	270	1.00	1.00, 1.00	0.012	264	1.00	1.00, 1.00	0.040
**Treatment**								
Chemo	147	—	—		147	—	—	
ICI + Chemo	123	0.88	0.65, 1.20	0.4	117	0.67	0.48, 0.92	0.015
**RT**								
no	240	—	—		234	—	—	
yes	30	0.73	0.45, 1.17	0.2	30	0.70	0.41, 1.17	0.2
**Surgery**								
no	264	—	—		258	—	—	
yes	6	0.89	0.36, 2.17	0.8	6	0.97	0.36, 2.62	> 0.9

Abbreviations: CI = Confidence Interval, HR = Hazard Ratio

**Fig. 3:**
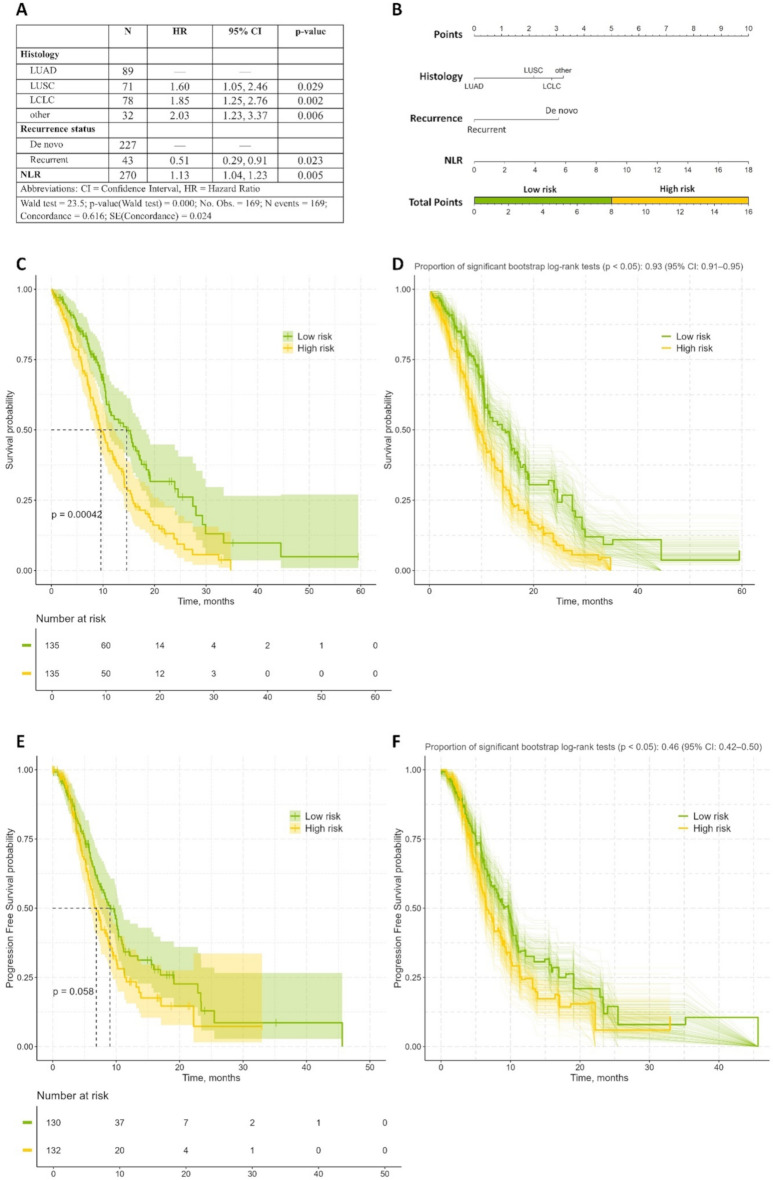
Multivariable Cox model development and risk stratification for NSCLC patients. **(A)** Multivariable Cox proportional hazards model. Table displaying hazard ratios (HR) with 95% confidence intervals (CI) and *P* values for predictors of overall survival (OS) in NSCLC patients. **(B)** Nomogram for risk calculation: total points (median cutoff) stratify patients into low-risk (green) and high-risk (yellow) groups. **(C)** Kaplan–Meier curves of OS in the low- and high-risk groups. Censored data marked as vertical ticks. **(D)** Bootstrap validation of survival analysis. Distribution of 500 bootstrapped Kaplan-Meier curves (thin lines) with average survival estimates (bold lines) for risk groups. Proportion of significant log-rank tests (*p* < 0.05) = 93.4% (95% CI: 90.8–95.2%) confirms the robustness of risk stratification. **(E)** Kaplan–Meier curves of progression-free survival in the low- and high-risk groups. Censored data are marked as vertical ticks. **(F)** The bootstrap validation of survival analysis. Distribution of 500 bootstrapped Kaplan-Meier curves (thin lines) with average progression-free survival estimates (bold lines) for risk groups. Proportion of significant log-rank tests (*p* < 0.05) = 93.4% (95% CI: 90.8–95.2%) confirms the robustness of risk stratification

Variables with a significant level of *p* < 0.1 were retained for further analysis. The backward elimination process was used to determine the final multivariate Cox model. Histology, recurrence following curative treatment, and NLR variables were included in the final multivariate Cox model (Fig. 3A).

The model satisfied the proportional hazards assumptions (global Schoenfeld test *p* = 0.53), with no influential outliers and non-linear effects for continuous variables (Figure S1). The model demonstrated moderate discrimination (Harrell’s C-index = 0.616, SE = 0.024). Nevertheless, bootstrapped calibration plots demonstrated a high degree of concordance between predicted and observed 12- and 24-month survival probabilities (slope = 0.854; 0.846) (Figure S2).

### Risk stratification and treatment effects

Based on the final multivariate Cox model, a clinical nomogram was created to translate the model coefficients into a clinically usable tool. According to the nomogram, a total risk score was calculated for each patient. Using the median risk score as a cut-off point, the patients were divided into high-risk (*n* = 135) and low-risk (*n* = 135) groups (Fig. 3B). Patients in the high-risk group had a significantly shorter OS (9.6 vs. 14.6 months, *p* < 0.001) and PFS (6.9 vs. 9.0 months, *p* = 0.058) compared with those in the low-risk group (Fig. 3C, E). The low-risk group comprised a higher proportion of females (28% vs. 16%), recurrent cases (32% vs. 0%), and adenocarcinoma histology (60% vs. 5.9%), while high-risk patients predominantly had stage IV disease (70%) and elevated systemic inflammation markers (NLR, PLR, MLR, *p* < 0.001 for all) (Table 3).

**Table 3:** The clinicopathological characteristics of low-risk and high-risk NSCLC patient groups. Histology: LUAD (lung adenocarcinoma), LUSC (squamous cell carcinoma), LCLC (large cell lung cancer). TNM (T/N/M): tumor, node, and metastasis staging classifications. “X” denotes unspecified staging. RT: radiotherapy. NLR/MLR/PLR: Neutrophil-to-lymphocyte, monocyte-to-lymphocyte, and platelet-to-lymphocyte ratios (continuous variables)

Characteristic	Low Risk *N* = 135^*1*^	High Risk *N* = 135^*1*^	*p*-value^2^
**Gender**			0.012
female	38 (28%)	21 (16%)	
male	97 (72%)	114 (84%)	
**Age**	64 (57, 71)	66 (60, 72)	0.13
**BMI**	25.1 (23.2, 28.7)	24.2 (21.0, 27.5)	0.005
**Histology**			< 0.001
LUAD	81 (60%)	8 (5.9%)	
LUSC	35 (26%)	36 (27%)	
LCLC	13 (9.6%)	65 (48%)	
other	6 (4.4%)	26 (19%)	
**TNM (T)**			< 0.001
1	18 (13%)	5 (3.7%)	
2	32 (24%)	16 (12%)	
3	39 (29%)	27 (20%)	
4	42 (31%)	82 (61%)	
X	4 (3.0%)	5 (3.7%)	
**TNM (N)**			0.040
0	24 (18%)	14 (10%)	
1	29 (21%)	25 (19%)	
2	49 (36%)	56 (41%)	
3	22 (16%)	36 (27%)	
X	11 (8.1%)	4 (3.0%)	
**TNM (M)**			0.039
0	60 (44%)	40 (30%)	
1	72 (53%)	92 (68%)	
X	3 (2.2%)	3 (2.2%)	
**Stage groups**			< 0.001
I-II	18 (13%)	1 (0.7%)	
III	43 (32%)	39 (29%)	
IV	74 (55%)	95 (70%)	
**Recurrence status**			< 0.001
De novo	92 (68%)	135 (100%)	
Recurrent	43 (32%)	0 (0%)	
**NLR**	2.45 (1.74, 3.32)	3.48 (2.54, 4.72)	< 0.001
**MLR**	0.38 (0.27, 0.49)	0.50 (0.37, 0.65)	< 0.001
**PLR**	143 (104, 197)	192 (139, 250)	< 0.001
**Treatment**			0.020
Chemo	83 (61%)	64 (47%)	
ICI + Chemo	52 (39%)	71 (53%)	
**RT**			0.7
no	119 (88%)	121 (90%)	
yes	16 (12%)	14 (10%)	
**Surgery**			0.030
no	129 (96%)	135 (100%)	
yes	6 (4.4%)	0 (0%)	

^*1*^n (%); Median (Q1, Q3)

^*2*^Pearson’s Chi-squared test; Wilcoxon rank sum test; Fisher’s exact test

The bootstrap internal validation, which was applied to assess result stability, confirmed robust risk stratification: 93% of resampled datasets (95% CI: 91–95%) replicated significant OS differences, and 46% (95% CI: 42–50%) replicated PFS differences (Fig. 3D, F).

### The effect of chemotherapy and combination therapy in risk groups

**Fig. 4:**
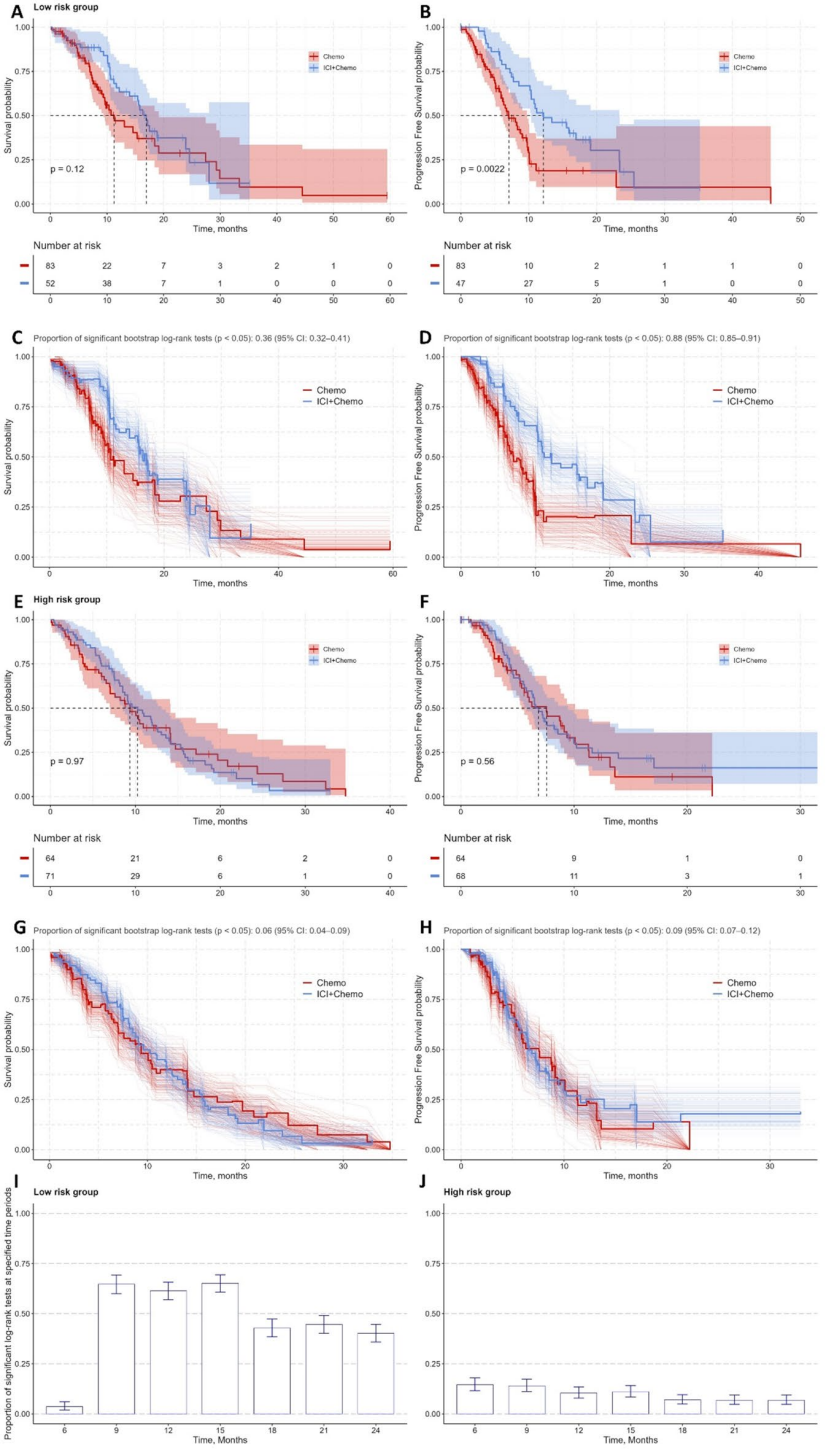
Risk-stratified survival analysis and treatment benefit validation in non-small-cell lung cancer patients. Panels A-B (Low-risk group): Kaplan-Meier curves for **(A)** overall survival (OS) and **(B)** progression-free survival (PFS) comparing ICI + Chemo vs. Chemo alone. **Panels C-D (Low-risk validation)**: Bootstrap internal validation (B = 500 iterations) of treatment benefit (ICI + Chemo vs. Chemo) for **(C)** OS and **(D)** PFS. **Panels E-F (High-risk group)**: Kaplan-Meier curves for **(E)** OS and **(F)** PFS in high-risk patients. **Panels G-H (High-risk validation)**: Bootstrap validation (B = 500) of treatment effect stability for **(G)** OS and **(H)** PFS. **Panels I-J** (Time-restricted survival analysis): Proportion of significant log-rank tests (*p* < 0.05) at predefined time points for **(I)** low-risk and **(J)** high-risk groups. Abbreviations: ICI = Immune checkpoint inhibitors; Chemo = Chemotherapy; OS = Overall survival; PFS = Progression-free survival

Survival analysis revealed divergent outcomes between risk groups treated with chemotherapy alone versus immunotherapy-chemotherapy combinations. In the low-risk group, combination therapy improved median OS (16.9 vs. 11.3 months, *p* = 0.12) and PFS (12.2 vs. 7.1 months, *p* = 0.002) compared to chemotherapy alone (Fig. 4A, B).

The bootstrap internal validation (B = 500) revealed that 36% of bootstrap iterations (95% CI: 32–41%) confirmed the statistically significant OS benefit, and the PFS improvement was consistent in 88% of iterations (95% CI: 85–91%) (Fig. 4C, D). This bootstrap methodology quantifies the stability of observed effects across resampled datasets, with higher percentages reflecting greater reproducibility. By contrast, high-risk patients derived no OS (10.3 vs. 9.4 months, *p* = 0.97) or PFS (6.9 vs. 7.6 months, *p* = 0.56) benefit from combination therapy (Fig. 4E, F), with fewer than 9% bootstraps supporting survival improvement (Fig. 4G, H). Time-dependent survival analysis revealed maximal OS benefit at 9–15 months in low-risk patients, with approximately 70% statistically significant bootstrap iterations showing statistical significance (Fig. 4I, J).

**Fig. 5:**
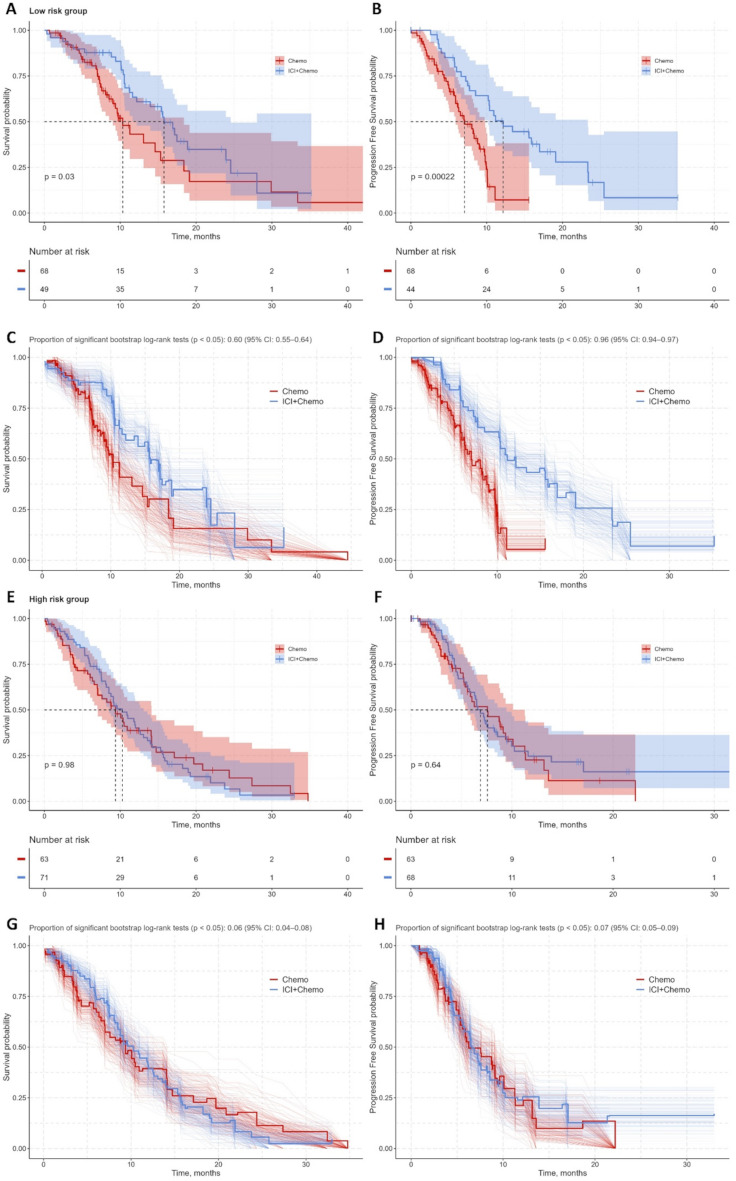
Risk-stratified survival analysis and treatment benefit validation in stage III-IV non-small-cell lung cancer patients. **Panels A-B (Low-risk group)**: Kaplan-Meier curves for **(A)** overall survival (OS) and **(B)** progression-free survival (PFS) comparing ICI + Chemo vs. Chemo alone. **Panels C-D (Low-risk validation)**: Bootstrap internal validation (B = 500 iterations) of treatment benefit (ICI + Chemo vs. Chemo) for **(C)** OS and **(D)** PFS. **Panels E-F (High-risk group**): Kaplan-Meier curves for **(E)** OS and **(F)** PFS in high-risk patients. **Panels G-H (High-risk validation)**: Bootstrap validation (B = 500) of treatment effect stability for **(G)** OS and **(H)** PFS. Abbreviations: ICI = Immune checkpoint inhibitors; Chemo = Chemotherapy; OS = Overall survival; PFS = Progression-free survival

Restricting the analysis to high-stage (III/IV) patients reinforced these findings. Among low-risk patients, the OS benefit of immunotherapy-chemotherapy was 15.8 vs. 10.3 months (*p* = 0.03), with this benefit persisting in 60% of bootstrap iterations (95% CI: 55–64%) (Fig. 5A, C).

By contrast, high-risk patients showed no meaningful OS gain 10.3 vs. 9.4 months (*p* = 0.98) (Fig. 5E, G). Similarly, combination therapy significantly prolonged progression-free survival in low-risk patients (12.2 vs. 7.1 months, *p* < 0.001) and remained significant in 96% of bootstrap iterations (95% CI: 94–97%) (Fig. 5B, D). Conversely, no significant PFS benefit was observed in high-risk patients (6.9 vs. 7.6 months; *p* = 0.64) (Fig. 5F, H).

In summary, the study identified a robust risk stratification model that effectively discriminates survival outcomes in NSCLC. Immunotherapy-chemotherapy provided significant PFS and moderate OS benefits in low-risk patients, whereas high-risk patients, characterized by aggressive biology and elevated systemic inflammation (NLR), derived minimal benefit, highlighting the requirement for alternative therapeutic strategies in this subgroup.

## Discussion

This study demonstrates the critical role of risk stratification in personalizing therapy for advanced NSCLC, leveraging a prognostic model incorporating histology, systemic inflammation (NLR), and recurrence status. The findings reveal distinct survival outcomes between low- and high-risk groups (OS: 14.6 vs. 9.6 months, *p* < 0.001). In the present cohort of patients with advanced NSCLC treated in a real-world setting, immunotherapy combined with chemotherapy conferred significant PFS benefit (12.2 vs. 7.1 months, *p* = 0.002) and a clinically meaningful trend in OS (16.9 vs. 11.3 months, *p* = 0.12) in low-risk patients, whereas high-risk patients derived minimal benefit (OS: 10.3 vs. 9.4 months, *p* = 0.97 or PFS: 6.9 vs. 7.6 months, *p* = 0.56).

Our RWD long-term survival data complement landmark large clinical trials demonstrating benefits with combinations of immuno-oncology (IO) and chemotherapeutic agents in advanced NSCLC. Nevertheless, the differential treatment effects observed across risk groups emphasize the limitations of a universal immunotherapy approach and raise significant questions regarding the selection criteria employed to define an IO-sensitive population [6–9, 13–17]. It is unfortunate that contemporary biomarkers, including PD-L1 expression, TMB, smoking status, single genetic alterations, and systemic inflammation, remain insufficient univariable parameters for reliably excluding IO non-responders. Consequently, clinical trials and guidelines include the set of broad clinical characteristics that facilitate the selection of a group of patients who achieve a statistically significant benefit in either endpoint, as opposed to defining one with a clinically significant benefit [18]. To address this gap, a multivariate model was developed on the RWD cohort of NSCLC with optimal first-line treatment. This model delineated groups with different prognoses that contained a sufficient number of patients with IO and chemotherapy-based regimens for comparison. To demonstrate the robust reproducibility of the model, internal validations via bootstrapping were employed. The model confirmed poorer survival in patients with large cell carcinoma (HR = 1.85, *p* = 0.002), lung squamous cell carcinoma (HR = 1.6, *p* = 0.03), rare histologic subtypes (HR = 2.03, *p* = 0.006), higher systemic inflammation based on the neutrophil to lymphocyte ratio (HR = 1.13, *p* = 0.005), and recurrence status (HR = 0.51, *p* = 0.023). Each of these parameters has been previously validated as a prognostic survival variable for NSCLC patients. Lung adenocarcinoma typically demonstrates a superior prognosis compared to squamous histology [19, 20]. Pre-treatment elevated NLR has been demonstrated to reflect a pro-tumor inflammatory microenvironment and to predict poor outcomes across NSCLC stages, potentially serving as a predictive biomarker for immunotherapy benefit [21, 22]. Patients with recurrent metastatic NSCLC demonstrate significantly better OS than those with de novo metastatic disease [23, 24]. Notably, early disease progression or lack of disease control during first-line therapy may serve as a surrogate marker of poor prognosis in subsequent treatment lines [25]. The combination of these prognostic variables in a multivariable model has been shown to offer a promising tool for the risk stratification of patients with NSCLC. Moreover, the incorporation of NLR as a continuous variable provides a more sensitive and biologically plausible reflection of the spectrum of systemic inflammation’s impact on survival outcomes. This avoids the arbitrary dichotomization inherent in cut-off-based approaches commonly reported in the literature [22]. The differential treatment effects observed across various risk groups suggest that this model could aid in tailoring treatment strategies. Additionally, the economic implications of personalized treatment selection extend beyond individual patient outcomes to encompass broader considerations within the healthcare system. Patients considered to be at high risk, who exhibit minimal benefit from combination therapy, may be eligible for sparing from the potential toxicity of checkpoint inhibitors and the significant financial burden associated with ineffective treatment. This consideration assumes particular relevance in healthcare systems with limited resources, where the cost-effectiveness of treatment regimens must be carefully evaluated.

Several limitations must be acknowledged in interpreting these findings. Firstly, the retrospective design has the potential to introduce selection bias and a heterogeneous patient population. Secondly, the exclusion of PD-L1 data is recognized as a predictor of the immunotherapy response. Thirdly, the relatively modest cohort size of NSCLC patients, particularly after risk stratification, the cohort, and the lack of external validation due to variable incompatibility in public datasets. Notwithstanding these limitations, the model successfully incorporates important prognostic clinical variables, effectively stratifies NSCLC patients into distinct risk groups, and demonstrates significant improvements in PFS and OS for low-risk patients receiving combination treatment. Future research should focus on the integration of molecular markers into the model and on their prospective validation as a means of guiding treatment decisions.

## Electronic supplementary material

Below is the link to the electronic supplementary material.

Supplementary Material 1

## Data Availability

No datasets were generated or analysed during the current study.
