# Supplementary material for: Prognostic model integrating histology, systemic inflammation, and recurrence status predicts immunotherapy response in advanced non-small-cell lung cancer patients
Source: Biol Direct. 2025 Jul 3;20:78. doi: 10.1186/s13062-025-00674-3 (PMC12224361; doi:10.1186/s13062-025-00674-3)
Supplement: Supplementary file 1 — Supplementary Material 1 [file 13062_2025_674_MOESM1_ESM.pdf]

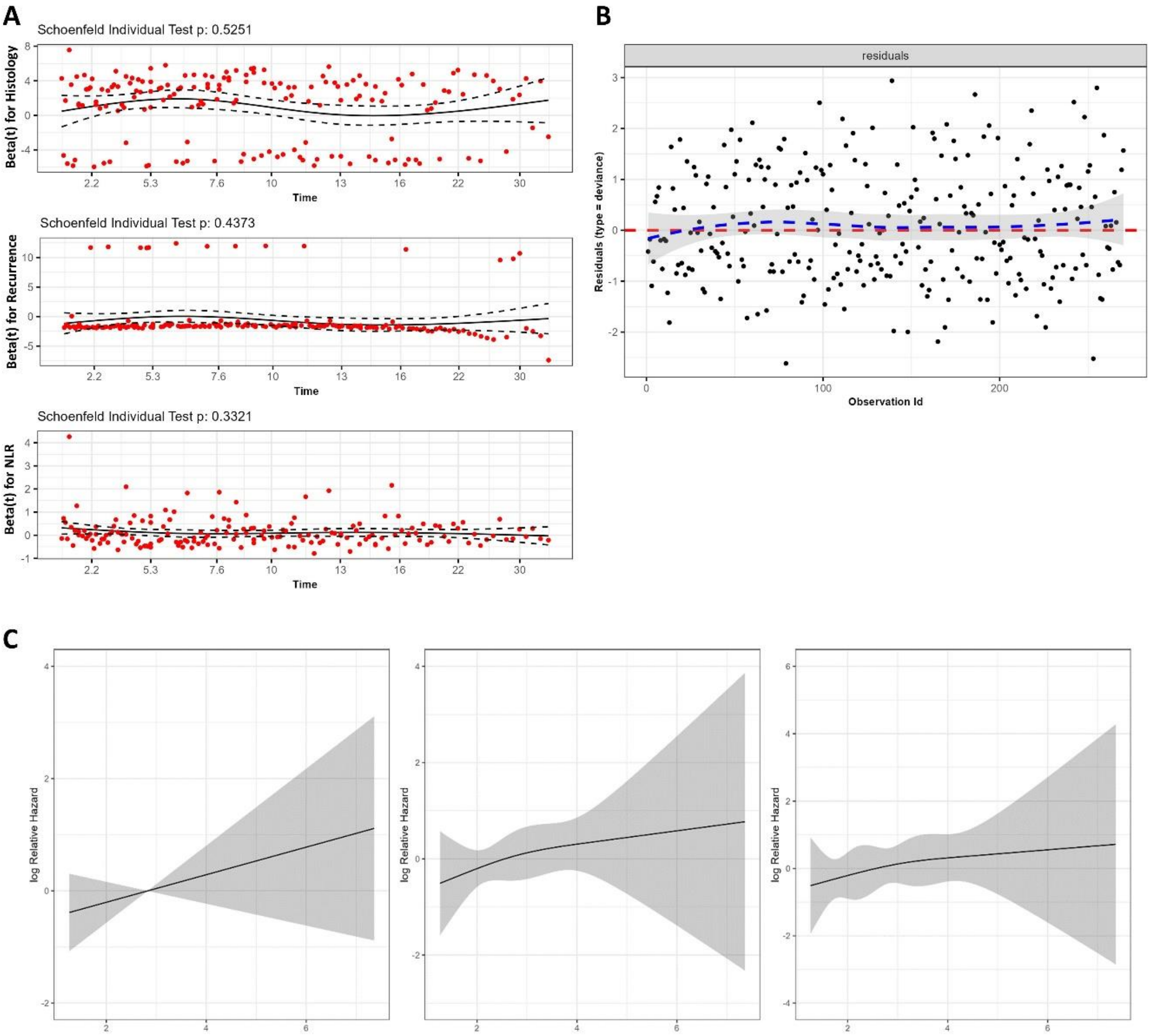

	Chi-Square	d.f.	P
NEU_LYMP	1.116	1	0.291
TOTAL	1.116	1	0.291

	Chi-Square	d.f.	P
NEU_LYMP	1.208	2	0.547
Nonlinear	0.092	1	0.762
TOTAL	1.208	2	0.547

	Chi-Square	d.f.	P
NEU_LYMP	1.223	3	0.747
Nonlinear	0.107	2	0.948
TOTAL	1.223	3	0.747

Figure S1. Diagnostic and non-linearity assessments for the multivariable Cox proportional hazards model. (A) Schoenfeld residuals plot testing the proportional hazards assumption. Deviations from a horizontal line suggest violations of the assumption. **(B)** Deviance residuals plot evaluating model fit and potential outliers. **(C)** Log-hazard predictions for NLR (solid lines: linear model; dashed lines: restricted cubic splines with 3 or 4 knots). Shaded regions represent 95% confidence intervals. Likelihood ratio tests (LRT) compared linear *vs*. spline models to evaluate non-linear associations.

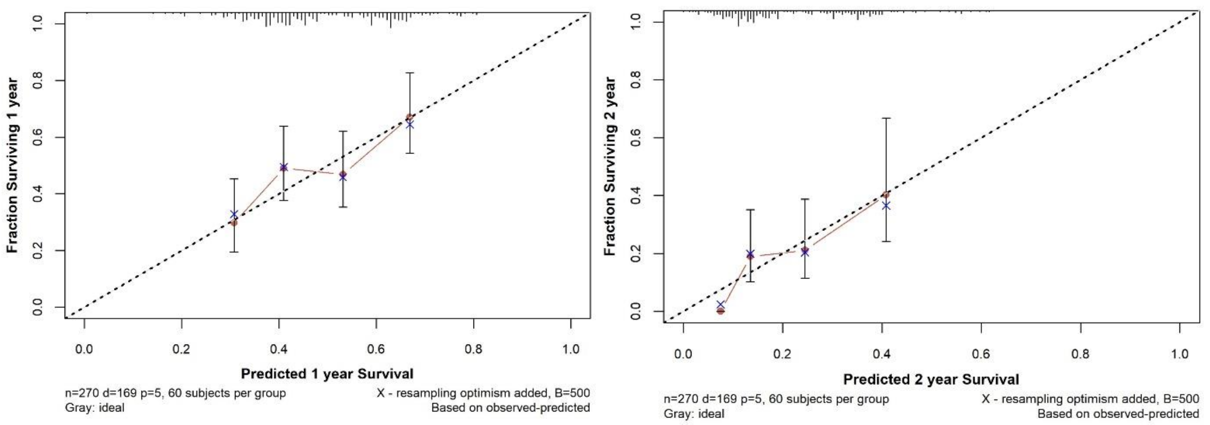

Figure S2. The bootstrapped calibration curves for 1-year and 2-year survival predictions. Predicted *vs*. observed survival probabilities (n = 270, events = 169). The dotted line represents ideal calibration.
